# The “One Health” approach in the face of Covid-19: how radical should it be?

**DOI:** 10.1186/s13010-022-00116-2

**Published:** 2022-02-28

**Authors:** Vittorio A. Sironi, Silvia Inglese, Andrea Lavazza

**Affiliations:** 1 Medical School, Bicocca University, Monza, Milan Italy; 2 Fondazione Irccs Ca’ Granda - Ospedale Maggiore Policlinico, Milan, Italy; 3 Centro Universitario Internazionale, Via Garbasso, 32, 52100 Arezzo, Italy; 4 Dipartimento di Scienze del Sistema Nervoso e del Comportamento, University of Pavia, Via Bassi, 21, 27100 Pavia, Italy

**Keywords:** Environment, Holistic view, Antibiotic resistance, Bioethics

## Abstract

**Background:**

The 2020-2021 coronavirus (Covid-19) pandemic is just the latest epidemic event that requires us to rethink and change our understanding of health. Health should no longer be conceived only in relation to human beings, but in unitary terms, as a dimension that connects humans, animals, plants, and the environment (holistic view, One Health). In general, alterations occurring in this articulated chain of life trigger a domino effect.

**Methodology:**

In this paper, we review the One Health paradigm in the light of the Covid-19 pandemic and distinguish two approaches within it that might be dubbed the Prudent one and the Radical one. Each approach is structured in three levels – epistemological, medical, and ethical.

**Results:**

In this way, we show how we humans can better address the pandemic today and how, in the future, we can treat the whole living system better, by renouncing our anthropocentric perspective on health.

**Conclusion:**

We hold that the Prudent approach can be very helpful, and we discuss the medical and ethical issues related to it. We also consider the Radical view and the epistemological turn it requires compared to the Prudent one.

## Introduction – Two “One Health” approaches

The 2020-2021 coronavirus (Covid-19) pandemic is just the latest epidemic event that requires us to rethink and change the current healthcare paradigms without any further delay. Health can no longer be conceived only in relation to human beings (anthropocentric view, human health), but should be considered in global terms, as a dimension that connects humans, animals, plants and the environment (holistic view, One Health) based on the fact that the health states of humans, animals, plants and the environment are interrelated in an evolutionary interpretation of the biosphere.

This paradigm shift may appear relatively simple to understand, as it seems to imply a view of health that has already been available for some time and appears in principle well founded. A more complex aspect, as will be seen, is the practical adoption of this paradigm and the acceptance of all its implications both at the individual level and at the social, political, and economic levels.

Today, there is no doubt that the alterations that occur in the first links of this articulated chain of life (the environment and the plant kingdom) inevitably trigger a domino effect: hence the pathological manifestations destined to affect, in the short or long term, animal as well as human health. This view, which inevitably leads to consider health in a unitary global perspective (One Health), is supported by the fact that there have been several outbreaks of new infectious diseases for some time now (one almost every year in the last five decades of our history) due to animal viruses via spillover, whereby they come to occupy ecological niches left free of other occupants. Together with the reappearance of serious bacterial infections caused by antibiotic resistance, this is leading to an epidemiological landscape of epidemic outbreaks and pandemic scenarios that previously seemed to have been overcome.

Medicine is currently able to rapidly develop effective preventive, diagnostic and therapeutic approaches (tests, drugs, and vaccines). However, the perspective we are discussing poses new (bio)ethical problems concerning the universality of care and the differences in terms of access to the new approach to health, since the new approach would probably be more expensive and not all the countries could implement it soon. Moreover, the very idea of One Health seems to imply cultural changes that should be supported from below and not imposed from above. The role and responsibilities of the various actors involved should therefore be assessed.

What we wish to note here, more specifically, is the fact that while many approaches grouped under the idea of ​​One Health can be useful for limiting the spread of new epidemics or pandemics, they face significant implementation difficulties because they require significant changes in current medical, economic, social, and even ethical practices. Also, they are not epistemologically different from the anthropocentric models that put human health hierarchically first.

In this framework, environmental ethics can profitably dialogue with health ethics, as the former provides a framework of theories relating to the protection of natural entities and the use of nature's resources. Environmental ethics [1, 2] represents a broader framework which can include the idea of One Health, insofar as the latter is ethically connoted. Arguments and theories put forward in environmental ethics can be used to assess the impact of humans on the planet, what kind of protection we should provide for it and for what purpose. The main competing paradigms at stake are anthropocentrism and ecocentrism, in which the place of human beings in nature is conceived differently. It must be remembered, however, that environmental pragmatism presents a third option which we shall briefly examine in the section on ethical issues.

The purpose of this article is therefore to argue that the One Health paradigm should always be clearly specified, so as not to place proposals under this label if they are not consistent with its original requirements. In this sense, we propose to distinguish between two types of One Health approach, which are connected to the more general framework of environmental ethics, as mentioned above. The first one will be called Prudential One Health Approach (POHA): it considers prevention and treatment in a broad perspective, but is always, even if indirectly, centered on the human being. The second one will be called Radical One Health Approach (ROHA): it considers the overall balance of the living eco-system and the environment from a broader perspective than the human one.

In our opinion, this distinction is crucial both from the analytical-scientific viewpoint and from the viewpoint of the policies that can be implemented following one model or the other. In particular, confusion between the two approaches, which have different implications and consequences from the ethical viewpoint, should be avoided. In general, it can be argued that the Prudential One Health Approach (POHA) is a significant step forward in the direction of the protection of human health as well as of some environmental aspects that would improve the conditions of some other living entities. The POHA should certainly be pursued.

On the other hand, the Radical One Health Approach involves a true epistemological and ethical change that appears neither easy to accomplish nor simple to implement through consistent policies. In the rest of this paper, we will try to address the epistemological, medical and ethical issues related to the One Health approach, trying to make the distinction we introduced explicit and straightforward.

### Epistemological issues

The One Health concept was introduced at the beginning of the 2000s, even if its holistic approach is not new [3]. In fact, this view summarizes an idea that has been known for more than a century – that is, that human and animal health are at least interconnected (or possibly interdependent) and bound to the health of the ecosystems in which they exist. This concept is envisaged as a collaborative global approach to understanding risks for human and animal health, including both domestic animals and wildlife, as well as the health of ecosystems as a whole [4].

As per the definition provided by the One Health Initiative Task Force [5], One Health implies "the collaborative efforts of multiple disciplines working locally, nationally, and globally, to attain optimal health for people, animals and our environment". In other and simpler words, “you cannot tell the story of human health separate from animal health or environmental health” [6].

So, this approach recognizes that human health is strictly linked to that of animals, plants, and the environment, as these are realms with which we continuously interact. This view originated in ancient medicine: at the onset of rational medical thinking, medicine turned to the study of nature to understand the conditions of health and disease [7]. In this perspective, it seemed normal to believe that human beings were dependent on the environment in which they lived and on other forms of life. In fact, Hippocratic Greek medicine considered the human being in its complexity, from an ecological, biological, and social perspective. It connected the body with climate, nutrition, age, and even psychological features. Health and disease were inconceivable outside the environmental context.

This unitary view of health has become increasingly important in recent years because many factors have affected the interactions between people, animals, plants, and the environment. For this reason, it has become apparent that one should protect the health not only of human beings, but also of the whole ecosystem and its components. Medicine should therefore change its epistemological stance. It has thus become urgent and inevitable to research the causes of diseases by investigating the relationships between the human organism and the physical-social environment.

We are all constantly interacting with the biosphere that surrounds us: with the animal world (our pets or the animals whose meat and products we eat), with the vegetable one (the plants and herbs that we grow for food or the flowers in our gardens) and with the inanimate one (the air we breathe, the water we drink, the stones we use to build our homes). Today the ecosystem is conceived as something that is outside our body, but microbiology has shown that in addition to this external environment (macrobiome) – so fundamental for the balance of the biosphere – our organism contains a great variety of internal ecosystems (microbiome) that are no less important for our health [8].

This new knowledge highlights once more how the human being is part of an uninterrupted biological chain that goes from the macroenvironments populated by animals and plants to the microenvironment filled with germs with which we live in perennial symbiosis, and which are essential for a healthy functioning of our organism. It is evident that any alteration of these macro- and micro-ecosystems affects the balance between them, giving rise to pathological phenomena that inevitably affect the human organism [7].

The point is that if we wish to avoid, reduce or at least be prepared to consciously face future serious infections like Covid-19, which are likely to occur in an epidemic or pandemic form, an epistemological turn that leads to a One Health perspective can no longer be postponed. Yet this approach seems to maintain a hierarchy of interests, prioritizing human beings over non-human animals, and the latter over plants and the environment.

For example, let’s consider a series of dramatic changes/accelerations occurring in key areas and processes of the Earth system. In particular, nine of them are deemed particularly relevant to health. They are: integrity of the biosphere; climate change; new biological entities; ozone reduction; ocean acidification; availability of clean water; changes in the earth system; changes in the atmosphere; changes in the biogeochemical cycle of nitrogen and phosphorus (cf. [9]). Each of them seem to be decisive for keeping the Earth intact as a whole, and some are particularly relevant in relation to the pandemic emergency.

Climate change and the destruction of the natural habitat in some areas of the world have pushed bats, considered the "reservoir" of corona viruses, to move to areas where they are in greater contact with humans and other species. Growing urbanization destroys “buffer zones” that act as natural barriers between humans and other species, increasing the opportunities for pathogens to escape. The association between air pollutants, the spread of Covid-19 and the severity of lung disease has also been highlighted, on the one hand due to the effect of pollution on the spread of infection; on the other hand, due to the aggravation of symptoms for those affected by the disease [10].

It is therefore evident, from an epistemological viewpoint, that the very choice of processes / situations that deserve attention implies a strongly anthropocentric perspective. In fact, we tend to consider the situations with a greater anthropogenic character and the processes that affect human health. In this sense, the One Health approach calls for an important "broadening" of the factors considered without, however, a real change in perspective, method and purpose of knowledge relating to health, such as to configure a radical epistemological shift.

From this point of view, POHA allows us to see aspects that were unclear until now. Among these elements, we can mention the role of other animal species in the spread of viruses, the effects of a development model that is not very sensitive to the environment, the direct and indirect impact of climate change... Instead, ROHA entails a shift from the perspective that makes us look at phenomena only in terms of the effects they have on us. ROHA seems to include the challenges of the Nagelian view from nowhere and that of the Smithian impartial spectator, but projected into a global and interspecies dimension.

For example, according to ROHA, climate changes have occurred throughout the history of the Earth and many species have become extinct, as it were, "naturally"; the food chain means that individuals of some species systematically perish whereas individuals of other species living in habitats where there are not predators are not threatened at all. Sea level rise primarily damages human settlements and, from the ROHA perspective, it is no different from the extinction of dinosaurs due to the impact of an asteroid (if that is the explanation for their extinction). Should we revive dinosaurs via genetic engineering and let the sea submerge Venice? This is not necessarily an unsolvable dilemma, but such an example shows how difficult it is to take a truly non-anthropocentric epistemological perspective.

Indeed, the dinosaur example shows well some of the difficulties faced by ROHA (cf. [11]). Firstly, why should we stop at dinosaurs? One way of reading ROHA is that all extinct life should be resurrected. But how could that be possible, since those forms of life require very specific ecosystems and we could not reproduce them all at the same time. So, we couldn't choose, if all life is equal after all. A more charitable reading of ROHA would be that we ought to protect all life as it currently exists. But is that understood at the individual or species level? In the latter case, the human population may have to dwindle and live very differently. Also, one might paradoxically wonder if pathogens have some moral status that requires keeping them going. Many humans would object, and for good reasons, but it would be easy, for instance, to keep SARS-CoV-2 alive in bats that are not affected by it.

Beever and Morar [12] interestingly distinguish between two models of One Health approach, namely the moderate one and the strong one. They propose to articulate the two approaches according to different dimensions. As for the ontological conceptual space, if the traditional approach to (human) health is "independent", the moderate One Health approach is "interconnected", and the One Health strong approach is "interdependent". From an epistemic point of view, while the traditional approach is "disciplinary", the moderate One Health approach is "multidisciplinary", and the One Health strong approach is "interdisciplinary".

This means that two paradigms implicitly coexist under the same One Health term. The first, which is based on the concept of *interconnection*, recognizes the existence of an inextricable link among human, animal, and environmental contexts [13]. This implies that "health and disease cannot be properly understood, and properly promoted or responded to, by a single discipline given the various domains of knowledge intersect in order to make sense of our organic complexity" [12].

The second paradigm focuses on *interdependence*, namely the view that the whole is constituted by the relationship among its parts. This view is opposed to the idea of interconnection, which entails that the universe is organized in discrete units that simply interact with one another. The interdependence model [14] involves an important shift of perspective. Take for example the case of the human microbiome:a microbial ecosystem consisting of thousands of billions of microorganisms - the intestinal microbial - whose total mass is about 0.2-1 kg in a person weighting 70 kg.

“This microbial view of human organisms, arguing that we each are co-constituted by the microbial community that makes us up, extends well beyond ourselves to include also the non-human animal and even the plant. At the level of human organism, there is a clear interdependence between the human organism and the microbial community that co-constitutes it: neither the host nor the microbial community would exist as such without interdependent reliance on the other’s biochemical and nutrient services, or at least they would not be able to thrive as healthy organisms” [12].

These two perspectives are often confused. In fact, scholars often seem to adhere to a perspective that combines elements of both. As we know, the classical, monodisciplinary perspective thought of human health as distinct and superordinate, a subject to be studied by a separate discipline, with its own epistemic status based on a specific ontology of the biological world. The transition to the One Health approach overcomes the cornerstones of that concept, introducing the overall consideration of the living world in the description and treatment of health.

This widening of the ontological and epistemic perspective on health is mainly based on the inclusion of the animal world, also due to the contingent fact that veterinarians were the protagonists of the turn towards the One Health model. But the implications of this shift can and should be deeper and more inclusive, according to the two models presented above. The distinction we made between POHA and ROHA partly embraces the partition proposed by Beever and Morar [12], but it seems to be more helpful as it highlights how the Prudential One Health Approach is not really in line with the basic ideas of ​​One Health and outlines its potential implications in general and in terms of policies.

Two clarifications need to be made here. First, we should specify what we mean by the living world and how we understand its functioning. Do we believe that the living world is made of distinct but interconnected biological entities or that it is an interdependent whole, in which it is possible to distinguish between entities only from an analytical point of view but not from a practical and ethical point of view? Second, it should be specified if our primary concern is still human health (although framed in the overall balance of a living world that must be considered in its entirety) which is more important than that of other living forms, or if instead we want to find a balance that does not favour our species at the expense of the entire ecosystem.

### Medical issues

The Covid-19 pandemic has surprised the world’s medical community, which didn’t know how to respond to it effectively for many months. This should not be considered a failure of health systems, but rather a time when medicine should question its certainties and traditional methods of dealing with infectious diseases. It is clear that more complex strategies are needed for similar cases: we need more than simple medicinal therapies (antivirals and drugs targeted for complications) or mass immunization (vaccination). In fact, these measures – as we are seeing with the Covid-19 pandemic – are not always immediately available or possible for new pathogens.

The One Health approach is of great relevance from this point of view. The assessment of the role that ecological change plays in bringing out new zoonotic infectious diseases (which pass from animals to humans) is crucial for the timely recognition and control of these pathological conditions. But, again, this is simply part of POHA, in that it implies a strong anthropocentric view with the aim to control the spread of viruses: the only goal is to spare humans from the disease.

As medical-biological knowledge progressed, the Eco-Health perspective was developed as a natural expansion of the germ theory and subsequently of the theory of viruses as infectious agents. So, ecological aspects were finally integrated in the medical view. The relevance of ecosystem health to human and animal health was fully acknowledged only some decades ago, when the One Health vision became the dominant approach to deal with emerging threats related to the avian flu pandemic [15].

The in-depth study of the relationships between the environment, the host and the infectious pathogen is fundamental to try to contrast the occurrence of these situations [16]. The health threat posed by zoonoses is growing rapidly due to several factors: in particular, the demographic boom, intensive farming, excessive agricultural exploitation, deforestation, and climate change.

A historically important contribution to the knowledge of these interconnections between human and animal health has been provided by the discovery of zoonotic diseases and their complexity. The evidence that an animal virus such as that of the plague could give rise to a deadly human virus such as that of measles was indeed a “revelation” [17].

In the domain of zoonotic diseases, there is a wide range of pathological conditions carried by bacteria, viruses, parasites, fungi and even prions [18]. The world health landscape of the last fifty years, in this regard, has been disconcerting and glum. Before the Covid-19 pandemic, the impressive list of the main infectious diseases that emerged since the 1970s was the following: HIV (AIDS) / Hemorrhagic fever from Hantavirus / Lassa fever / Marburg fever / Legionella pneumonia / Hepatitis C / Lyme disease / Rift Valley fever / Ebola / Nipah disease / West Nile virus / SARS (Severe Acute Respiratory Syndrome) / Spongiform bovine encephalopathy / Avian plague / Middle East Respiratory Syndrome (MERS) / Chikungunya / Norovirus / Zika Gastroenteritis [19].

These serious zoonotic diseases are due to spillover, i.e., the passage of the pathogen from animals to humans [20]. Furthermore, zoonotic pathogens easily undergo mutations after the species barrier jump, so as to facilitate their adaptation to hostile environmental conditions before their spillover onto humans. In the last two decades, we have been able to ascertain the rapid spreading capacity and the severity of mutagenic coronavirus pathologies, which have determined epidemic outbreaks with a high mortality rate, posing a significant threat to world public health [21]. Even the current Covid-19 pandemic, based on the analysis of the first known cases, perhaps originated in a wet market in Wuhan, China – that is, in a place where the coexistence of various wild animal species facilitates the spillover of zoonotic viruses, which thus become new pathogens for humans.

Nor can we completely rule out the possibility that SARS-CoV-2 was created in a lab - indeed, there may be similar anthropogenic pathogens in the future. A virus may be lab-engineered for military purposes, but the ROHA approach seems to impose a reconsideration also of what can and cannot be done for military purposes and related industrial interests. In this sense, ROHA is a very radical perspective, which does not only question environmental policies in the strict sense.

If, in the context of a global vision of health, no effective action is taken to change these situations of ecological promiscuity and socio-economic weakness, the risk of new epidemics and new pandemics will remain high in the coming years [22]. That is why, in the context of the One Health vision, it is essential to consider the conditions of human-animal-environment interaction that are favorable to the transmission of infectious viruses, in order to recognize and eliminate the natural reservoirs of the pathogens themselves.

This vision is fundamental to understand transmission cycles and seek prevention mechanisms in the context of international scientific and institutional collaboration. For several millennia, until the beginning of the twentieth century, microbes represented the main pathogenic elements affecting humans and animals. Today, instead, the role of connectors between human, animal and vegetable health is being taken on by viruses. There is a fully predictable biological circularity within the natural mechanisms of evolution underlying the life of living beings, including microorganisms, which medicine must learn not to neglect in the context of its cognitive analysis procedures, in order to avoid finding itself unprepared to face new pathological conditions that can affect human health [23].

In this sense, reducing human pressure on the environment becomes a wide-ranging medical intervention in its own right. Climate change with anthropogenic causes and the anthropization of new territories pushes many species to move so as to find better conditions in new habitats. In the case of the recent coronavirus epidemic, it can generally be argued that if we humans eat the animals that host the viruses and interfere with the animals that act as reservoirs with these pathogens, as said above, diseases will spread widely [24]. The use of pesticides also forces pathogenic microorganisms to exploit their natural variability and change, making it more difficult to find the right cure for new variants of the viruses themselves [25, 26].

At the same time, another global emergency – comparable to that of climate change – is antibiotic resistance: a phenomenon that indicates that many pathogenic bacteria are no longer sensitive to the antimicrobial drugs available today. These microbes become dangerous because they can suddenly acquire entire sets of genes for antimicrobial resistance from completely different types of bacteria through horizontal genetic transfer. That is why the problem of multi-drug resistant microorganisms has spread so rapidly in the world. Such discoveries require us to modify our fundamental ways of understanding who we are as a human species, what has contributed to make us evolve and on which interconnections the functioning of the living world is based.

An interesting example is given by the history of colistin, a molecule discovered in the 1950s. Although effective, it was soon abandoned for use in humans due to a number of side effects, so it was used mainly on animals. In recent years, however, due to the increase in drug resistance, the use of colistin has been re-evaluated for humans and has instead been severely reduced in farm animals [27, 28]. This shows how many clinical choices at an individual patient’s bedside can be linked to what happens in the long chain of the One Health approach, and how strongly pharmacology itself is strongly interconnected. In this sense, new health policies should favor vaccination for the main diseases affecting farm animals instead of treatment with antibiotics. It is prevention that promotes the effectiveness of antibiotics when they really are necessary.

Super-microbes - selected over decades of antibiotic treatments, often used in excessive or inadequate ways in human medicine and for non-therapeutic reasons in the veterinary field -  are now a real threat and difficult to control. They are the result of the circulation and recirculation between human beings and their surroundings [29, 30]. The environment, as well as contact with pets, can represent an important source of contagion.

Just think of the dramatic problem of hospital infections that cause several tens of thousands of deaths worldwide every year. These infections are spread in both developed and developing countries. According to a recent World Health Organization (WHO) estimate, about 15% of all hospitalized patients suffer from such infections. These should be contrasted with specific prevention strategies (hand washing and sanitization of environments) and therapy (targeted use of antibiotics with rapid recognition through the study of the genome of multi-resistant bacteria) [31].

Today, however, thanks to big data and artificial intelligence, medicine can better deal with these insidious infections. Yet a different paradigm is also needed in the health field. Collecting and analyzing a huge amount of data – which are not only related to humans but also to animals, plants and the environment – for the first time in history allows us to have information about the whole global ecosystem and the possible health implications of any changes that may occur in it. With artificial intelligence it is possible to know and compare an impressive amount of clinical data that can help formulate more precise diagnoses, hypothesize adequate preventive scenarios and identify effective therapies against already known diseases, but above all against pathological conditions still unknown [32].

In these situations, POHA allows us to exploit the global information available in order to preventively protect the human being’s health insofar as it is influenced by a myriad of factors that, without an epistemological and clinical readjustment and without new technologies, we could not consider in their entirety. This epistemological and clinical readjustment has implications for other forms of life and the environment in which they live to the extent that they affect the human being. The fight against climate change or a limitation on the exploitation of certain natural resources is functional to human well-being but can have positive repercussions in general.

The case of ROHA is different: according to it, the epistemological and medical shift should be stronger. In this scenario, a completely non-anthropocentric perspective should be taken into consideration with the aim of rebalancing, as far as possible, the interests of all entities to which we can recognize a moral status, such as many animal species. In this sense, it should be emphasized that the SARS-CoV-2 virus appears to be harmful mainly to humans but not to other species, not even to the host individuals responsible for the initial infection.

### Ethical issues

The cognitive advance that can lead us to conceive and embrace POHA certainly will bring important consequences not only from the epidemiological viewpoint, but also from the ethical one. However, one cannot underestimate the fact that the holistic approach to health can also lead to two negative outcomes, even if only as unintended side effects. One is the risk of increasing inequalities in health protection [33], the other is to introduce an imperialist attitude [34]. Let us have a closer look at both points. Next, we will address the potential ethical implications of ROHA.

As for the first risk, at the national level, the increase in inequality may arise from the fact that POHA implies the availability of: (1) advanced knowledge (both in the form of scientists trained in this perspective and in terms of adequate scientific and health infrastructure); (2) economic resources (both as a domestic product and as additional funds allocated to research and health care for the purpose at hand) and, no less important; (3) willingness on the part of policymakers to adopt such a structural health policy. In the absence of even one of these elements, it is difficult for a country to make progress in implementing the new approach [35].

This means that less developed countries will find it more difficult to implement policies that appear costly, seem capable of delivering results only in the medium to long term (i.e. with an investment of resources that does not give immediate returns) and may also be unpopular from the point of view of political consensus for the leading groups, as has often happened with environmental policies in their initial phase. This slowness of action in less developed countries may mean that certain parts of the world at some stage will enjoy the benefits of POHA, while others will remain linked to less effective approaches to health, thus widening inequalities in this crucial area for people's lives.

On the other hand, POHA is, by definition, not confined to a single country. An interconnected and globalized world like ours – the Covid-19 pandemic has shown this very well – simply cannot be only *partially* secured against the viral threats arising from environmental imbalances. It is sufficient for one area to be fertile ground for the emergence of new pathogens for the whole of humanity to be potentially put at risk. Hence the danger of an imperialist attitude.

Indeed, this systemic environmental rebalancing might be carried out on a global scale by the most developed countries and imposed on the countries that have not yet embraced it. On the one hand, the One Health approach could constitute a form of international cooperation and aid, capable of benefiting all participants in the process, and specifically those who, as we have said, do not have the resources to face climate change on their own. On the other hand, however, there is a risk that this rebalancing action will be conducted from outside, without considering the traditions, beliefs, social customs, and habits of the populations involved.

The case of wet markets in East Asia can be an example in this respect. Wet markets and everything that revolves around them are part of an ancient and deeply rooted popular culture that cannot be easily erased with the prohibition of all the food practices related to them, as demonstrated by the interdictions introduced in the past only to be soon abolished [36]. There are obviously good reasons to regulate wet markets,in particular, the treatment of animals in such places does not meet the now widely recognized minimum ethical standards. However, imposing drastic regulatory changes in these areas can lead to strong resistance, which may result in lower compliance with the One Health approach.

Now, this is only one example and certainly not the most important one that could be made. In fact, more generally, the pressure from some countries on others to adopt specific rules in environmental and health fields – rules which are in everyone's interest but not yet generally understood and appreciated – implies an attitude that is at least paternalistic, if not imperialistic. This happens whenever there is an imposition in the form of conditional aid or other political pressures, for example in terms of supplies or access to markets that are subordinate to the acceptance of given health rules.

In this sense, the WHO and other supranational organizations at the regional level, such as inter-state associations or free trade areas, can play a key role here. In such forums, common procedures can be introduced to initiate the transposition and implementation of the One Health vision. In the same way, the international scientific community and major NGOs can play a role in fostering a cooperative, non-tax-based bottom-up approach. More developed countries, on the other hand, have a responsibility to lead the process and contribute to its implementation by bearing at least part of the costs for countries that do not currently have the resources to carry it out on their own [37].

Another set of ethical issues, as mentioned in section 2, related to epistemological (and ontological) issues, has to do with the distinction between two paradigms of the One Health approach. On the one hand, we have a Prudent Approach to One Health, which stresses the distinction of natural entities and their interconnection; on the other hand, we have the Radical Approach to One Health, which emphasizes the unity and interdependence of the natural and living world, highlighting the need to adopt a non-anthropocentric view. Both approaches differ from the traditional approach to health, which only considers the ontological and epistemic independence of the human race and seeks its flourishing, from a standpoint of an explicit ethical anthropocentrism.

When one goes beyond the individualistic concept of health to shift to a generic One Health approach, one can well do so for purely instrumental reasons. In this sense, as in the case of the COVID-19 pandemic, one can try to find causes and implement preventive methods by widening the public health perspective to animals and the environment, but with the sole purpose of safeguarding human health. Mass culling of infected or potentially infected animals is part of this approach.

However, this modality is reductive and is likely to be ineffective. In the first place, it seems to be only reactive, that is, it is concerned with the global balance of the natural world only when the human being is under threat, therefore often when it is already too late to act effectively. Secondly, it is an inconsistent and unsustainable long-term approach. In fact, if one adopts a One Health perspective, it is difficult to exclude some consideration for the welfare of non-human animals, and of the environment in general, both because the welfare of the human being also depends on environmental balance, and because we cannot overlook the suffering we inflict on other living species, given the scientific knowledge and moral sensitivity we have developed.

In this light, ROHA implies ecological egalitarianism as one of its assumptions. This means, in principle, that we should not give precedence to any one element of the natural world, all elements being equal from the moral point of view. This approach can be assimilated to the "deep ecology" proposed by Naess [38], in which all elements have the same value. Some have noted that in this perspective it is very hard to establish a hierarchy of interests and values in specific situations: if everything must be preserved in nature, not even the One Health approach can give us a definitive answer when, for example, we face a pandemic that affects humans after starting with non-human animals.

In this sense, the idea of interdependence implies an egalitarian ecological sensitivity that is not shared (yet) and therefore does not seem feasible from an ethical viewpoint, although it can remain a good guide for certain aspects of the research and the conservation of specific habitats. The radical approach requires consistent ethical choices that go beyond current animal rights ethics. For example, one might think that humans should leave a substantial part of the planet untouched so as to recreate a natural habitat for other species. Humans should also stop exploiting non-human animals, primarily for food, for example by resorting to cultured meat - something that now seems technologically feasible. The question remains as to how one could ethically act to prevent and fight pandemics, given that the ROHA places us on an equal footing with the rest of the living world.

Environmental pragmatism could offer a third option between ROHA and POHA, one where the former is the means to achieve the latter. Environmental pragmatism has been proposed by Norton [39]. The main claim of the theory, which is embedded in environmental ethics, is to deny that it is even necessary to choose between an anthropocentrist and a nonanthropocentrist ethics. The first reason why environmental pragmatism should not enter into this, and all related disputes is that what matters today is concrete action to influence (political) decision-making processes in an attempt to preserve the planet. Getting lost in theoretical discussions of principle risks doing more harm than good. Secondly, the division between anthropocentrists and non-anthropocentrists is based on an insufficiently deep concept of 'human interests' or 'human utility' that does not advance the discussion.

The environmental ethics championed by Norton should instead privilege a weak anthropocentrism, which “makes available two ethical resources of crucial importance to environmentalists. First, to the extent that environmental ethicists can make a case for a world view that emphasizes the close relationship between the human species and other living species, they can also make a case for ideals of human behavior extolling harmony with nature. These ideals are then available as a basis for criticizing preferences that merely exploit nature. Second, weak anthropocentrism as here defined also places value on human experiences that provide the basis for value formation” [40].

In this vein, the ROHA seems to ignore that fact, while the POHA generalizes anthropocentrism to an extreme. Therefore, a plausible pragmatist approach would be to expand the moral community beyond humans, doing so in a way that recognizes the diversity not only of life but of human values. A more eco-centric value system is still a human-centered approach, but it's not an approach that all humans currently endorse. A radical reform would include this value shift, which in turn would involve a radical reconsideration of what it means to be human. Anthropocentrism as it appears in the POHA retains a form of chauvinism that a radical ecological conception of human beings would at least curb.

However, in our opinion, it is more realistic to rely on a perspective of interconnection (POHA) in which the entities of the natural world have their own individuality and can be morally evaluated in themselves, although for the most part humans cannot thrive as a species except in a positive and respectful relationship with all other living species and the environment in a general sense. The risk in this case is that of returning to a full anthropocentrism typical of the traditional perspective on health. In this respect, a helpful warning comes from a group of scholars who recently wrote: "[One Health] arguably calls for an ethical framework that fully appreciates the moral value of biodiversity and environmental health beyond their mere instrumental value to human health" [41].

According to Capps and Ledermann [42], animals and humans are "inextricably linked" and "grounded in an ecological system that we share". The One Health perspective, therefore, does not consider this interconnection only as a heuristic tool for improving human health, but takes on its ethical significance and advocates an effort to improve the well-being of the human race (today and for future generations) along with that of other species and general ecosystems.

One Health, both as POHA and ROHA, can be more than a slogan only if we seriously consider all the implications of a perspective that is no longer only anthropocentric. However, a realistic position, which avoids the positions of the strong approach, which are currently held by a minority of people and are therefore considered extreme, seems so far to be more helpful and effective when it comes to bringing us into a new era of medicine and public health.

## Conclusion

In the light of the ongoing uncontrolled devastation of the environment, a new unitary view is needed. We should change our habits at all levels: reduce pollution, stop the destruction of the planet's green reserves, and put an end to the overexploitation of living organisms to limit the continuous erosion of biodiversity. Only the human species has both the ability and the responsibility to reverse this trend before it is too late. In this sense, we should go back to thinking about health in a broader and more global perspective, with a more forward-looking and not overly specialized approach.

We should learn to live with infections, epidemics and pandemics as it was done in past centuries, while trying to make the best use of the resources that medicine is able to offer us today: 1) extensive and proper use of vaccinations; 2) proper use of antimicrobials; 3) rediscovery of simple but often underutilized hygiene rules; 4) more ecological management of the environment; 5) increase in health protection in less developed countries.

Switching to a Prudent One Health Approach can greatly enhance the protection of our health and simultaneously improve the state of the planet. But only the adoption of a Radical One Health Approach will lead to the protection of the living ecosystem on an equal and not just anthropocentric level. A turning point of this type, however, still seems far away as it is our task as humans to change our epistemological perspective without someone else urging us to do so. A new sensitivity will have to arise from within, and new balances can only be achieved when the idea of ​​a ROHA has spread and established itself.

## Data Availability

Not applicable

## Abbreviations

POHA: Prudent One Health Approach
ROHA: Radical One Health Approach
WHO: World Health Organization
